# The Central R. R. Surgeons’ Association of Georgia

**Published:** 1892-05

**Authors:** 


### THE CENTRAL RAILROAD SURGEONS' ASSOCIATION OF GEORGIA.

The Central Railroad Surgeons' Association held their annual meeting in Columbus, April 19th.

The President, Dr. Seth N. Jordan, of Columbus, called the meeting to order and received the association in a few words of cordial welcome. The roll call showed that thirty-four members were present. After the minutes of last year's meeting had been read and confirmed, the program was carried out, with the exception that the essay on Anæsthetics, which was to have been read by Dr. Howard J. Williams, of Macon, was omitted, as Dr. Williams was unable to attend the convention.

Dr. W. H. Elliott, the chief surgeon of the Central system, was the first speaker introduced to the audience. He delivered a most interesting address on railway surgery, during which he treated in a very instructive way the relative merits of chloroform and ether.

Dr. S. N. Jordan followed Dr. Elliott in an interesting talk on when to operate.

Dr. A. C. North read a paper on his experiences in the treatment of railway injuries.

Dr. C. H. Richardson followed with a paper on Antiseptics in Railway Surgery. (See p. 268.)

The election of officers for the ensuing year was announced. The election resulted in the choice of: Dr. A. C. North, President; Dr. E. A. Perkins, Vice-President; Dr. H. B. McMaster, Secretary.

A committee on constitution was then appointed as follows: Dr. R. H. Taylor, of Griffin; Dr. N. G. England, of Cedartown; Dr. C. H. Richardson, of Montezuma.

The secretary requested the names of the surgeons who intended being present at the meeting of National Railway Surgeons' Association, which will be held at Old Point Comfort, the latter part of May. The following names were handed in: W. L. Felts, G. A. Bunch, J. S. Beard, J. P. Roblins, H. R. Mooney, R. M. Butts, W. H. Philpot, W. B. Nott, T. E. Jenning, A. C. North.

The meeting then adjourned for the year.

